# Functional and Numerical Responses of *Harmonia axyridis* (Coleoptera: Coccinellidae) to *Rhopalosiphum nymphaeae* (Hemiptera: Aphididae) and Their Potential for Biological Control

**DOI:** 10.3390/insects15090633

**Published:** 2024-08-23

**Authors:** Chong Li, Jingya Yu, Runping Mao, Kaili Kang, Letian Xu, Mengting Wu

**Affiliations:** 1 Anhui Province Key Laboratory of Forest Resources and Silviculture, Anhui Province Laboratory of Microbial Control, Engineering Research Center of Fungal Biotechnology, Ministry of Education, School of Forestry & Landscape Architecture, Anhui Agricultural University, Hefei 230036, China; 2 State Key Laboratory of Biocatalysis and Enzyme Engineering, School of Life Sciences, Hubei University, Wuhan 430062, China; 3 Institute of Plant Protection, Wuhan Institute of Landscape Architecture, Wuhan 430022, China

**Keywords:** biological control, predation ability, function response, predator–prey interactions

## Abstract

**Simple Summary:**

The aphid species *Rhopalosiphum nymphaeae* is a highly polyphagous pest that poses a significant threat to global agriculture. Biological control using natural enemies, such as the harlequin ladybird (*Harmonia axyridis*), is important for managing this pest. Our study evaluated the predation ability of *H. axyridis* in controlling *R. nymphaeae*. We examined the functional and numerical response of larva and adult *H. axyridis* preying on *R. nymphaeae*. Our results showed that the predation rate of individual *H. axyridis* on *R. nymphaeae* nymphs significantly increased as prey density increased. It was noted that fourth instar and adult *H. axyridis* were identified as optimal developmental stages in controlling *R. nymphaeae*. In addition, variations in intraspecific interference and self-interference exhibited by *H. axyridis* under increasing predator density were observed.

**Abstract:**

The water lily aphid (*Rhopalosiphum nymphaeae*) is a highly polyphagous herbivore that causes severe damage to many terrestrial and aquatic plants, especially lotus. Due to environmental concerns about water pollution and other issues caused by chemical control methods, there is an urgent need to develop effective and sustainable control methods. The harlequin ladybird (*Harmonia axyridis*) is a well-known aphid predator and may pose a potential threat to *R. nymphaeae*. To study the predation ability of *H. axyridis* at different developmental stages on *R. nymphaeae*, we assessed the functional response, attack rate, and search effect of *H. axyridis* larvae and adults preying on *R. nymphaeae*. The numerical response of this process was also evaluated under a constant ladybird-to-aphid ratio and constant aphid density conditions, respectively. Our results showed that all predator stages exhibited type II functional responses. The predation rate of individual *H. axyridis* on *R. nymphaeae* nymphs significantly increased as prey density increased. In contrast, the search effect of *H. axyridis* gradually decreased with an increase in prey density. Meanwhile, *H. axyridis* at different developmental stages possess varying predation abilities; fourth instar and adult *H. axyridis* were found to be highly efficient predators of *R. nymphaeae*. *H. axyridis* adults exhibited the highest predation ability and predation rate, while both the adult and fourth-instar larvae exhibited the highest attack rate. Moreover, fourth-instar larvae exhibited the highest search effect value at initially lower prey densities, although adults surpassed them at higher prey densities. Our results also indicated that *H. axyridis* exhibited varying degrees of intraspecific interference and self-interference influence as predator density increases. These results strongly support *H. axyridis* as an effective biocontrol agent for *R. nymphaeae*.

## 1. Introduction

The generalist herbivore, *Rhopalosiphum nymphaeae* (Linnaeus) (Hemiptera: Aphididae), is a highly polyphagous species that occurs on a large variety of terrestrial and aquatic plants, including American lotus, water hyacinth, water lettuce, water spinach, common duckweed, rice, and other cultivated crop and fruit plants [1,2,3]. Due to its high reproductive rate and short developmental time, this pest can rapidly increase its population size [4], posing a significant threat to global agriculture [5,6]. In addition, *R. nymphaeae* also transmits several viral diseases, such as brinjal mosaic virus, cabbage black ring spot virus, cauliflower mosaic virus, abaca mosaic virus, cucumber mosaic virus, and onion yellow dwarf virus, leading to die-back of water lettuce [7,8,9]. In China, *R. nymphaeae* has caused great damage to various plants, especially the Asian lotus, which holds cultural significance and is harvested as food. Effective management strategies for this pest are urgently needed.

Chemical control has been widely used to manage *R. nymphaeae*. Various types of pesticides, including neonicotinoid insecticides (imidacloprid, acetamiprid), agricultural antibiotics (avermectin), alkaloids (matrine), heterocyclic insecticides (pymetrozine), and other insecticides (cyantraniliprole) were found to be effective in reducing crop damage caused by *R. nymphaeae* [10,11]. However, the unsustainable nature and environmental toxicity of pesticides make them unwelcome in modern agriculture [12]. The water pollution resulting from pesticide use leads to the death of fishes, mollusks, arthropods, and other organisms, which has a significant impact on human health and the environment [13,14]. Therefore, considerable attention needs to be paid to effective and sustainable control methods for successful pest regulation [15]. Utilizing and releasing natural enemies for biological control is of great importance.

*Harmonia axyridis* (Pallas) (Coleoptera: Coccinellidae), commonly known as the harlequin ladybird, is a significant natural predator of many pests, especially aphids [16]. *H. axyridis* has become a prominent natural enemy that is highly regarded as a valuable component of integrated pest management due to its broad predatory capabilities, well-established application techniques, and extensively studied functional response [17,18,19]. Additionally, *H. axyridis* shares habitats with *R. nymphaeae*, which provides crucial baseline information for utilizing *H. axyridis* in the biological control of *R. nymphaeae*. It is, therefore, important to assess the predation ability of the natural predator *H. axyridis* on the polyphagous pest *R. nymphaeae* to develop effective biocontrol strategies in the field.

Measuring functional responses is essential for understanding predator–prey dynamics. This helps in describing how predation changes with variations in prey abundance and supports the development of effective and sustainable biocontrol programs [20,21]. Three typical types of functional responses have been identified based on how predation rates change as prey density increases [22]. Type I shows a linear increase in prey consumption with rising prey density until a maximum is reached, typically associated with filter feeders [23]. Type II demonstrates a negative density-dependent relationship, where the predation rate decreases at higher prey densities and is depicted by a hyperbolic curve. Type III describes a positive density-dependent relationship, where the predation rate increases at lower prey densities and then decreases at higher densities, resulting in a sigmoidal curve. Holling’s [24] classical functional response models include the predator’s attack rate (i.e., instantaneous search rate) and the handling time required to ingest prey. The attack rate measures the area of prey cleared per predator per unit of time while handling time refers to the time that the predator spends in attacking, killing, subduing, and digesting the prey [25]. High attack rates and low handling times indicate effective biocontrol agents. Predator individuals frequently encounter each other, and interference competition, including direct interactions, such as attacking conspecifics or exhibiting threat behavior [26], may decrease the consumption rate [27,28].

In this study, we evaluated the effectiveness of native natural enemy *H. axyridis* in managing *R. nymphaeae*. We examined the function response, attack rate, and search effect of individual larvae and adult *H. axyridis* (first, second, third, and fourth instars and adults) preying on *R. nymphaeae*. Furthermore, for analyzing the impact of intraspecific interference and self-interference, we assessed the numerical response of *H. axyridis* on *R. nymphaeae* under a constant ladybird-to-aphid ratio and constant aphid density conditions, respectively.

## 2. Material and Methods

### 2.1. Collection and Rearing of Ladybird and Aphid

The original population of *H. axyridis* (around 200) was collected from the Wuhan Institute of Landscape Architecture, China (GPS location: 30°6′ N, 114°4′ E) in 2022 and reared in the laboratory on *Rhopalosiphum padi* (Linnaeus) (Hemiptera: Aphididae), the wheat aphid, throughout the study. All ladybirds were collected at the same developmental stage after hatching or molting, and the female adults were collected before the oviposition period for subsequent experiments. The ladybirds were reared at 25 ± 1 °C with a relative humidity of 50–60% and a 16 h:8 h (light/dark) photoperiod.

The *R. nymphaeae* nymphs were collected from the lotus pond inside Wuhan Institute of Landscape Architecture, China (GPS location: 30°6′ N, 114°4′ E) from April to July. The 3rd and 4th instar nymphs of *R. nymphaeae* were used as prey in all experiments. All experiments were conducted in the same climatic controlled chamber under 25 ± 1 °C with a relative humidity of 50–60% and a 16 h light/8 h dark photoperiod throughout the rearing period.

### 2.2. Predation Ability of H. axyridis on R. nymphaeae Nymphs

The nymphs of *R. nymphaeae* in their 3rd to 4th instar were placed in a 7 cm diameter, 8 cm height plastic cup at room temperature. Moistened cotton wool balls were used to provide water for the nymphs. Before the experiment, all developmental stages of *H. axyridis* larvae (1st instar, 2nd instar, 3rd instar, 4th instar) and adult ladybirds were starved for 24 h. A single *H. axyridis* was then placed into each plastic cup. The densities of *R. nymphaeae* nymphs per cup were as follows: 6, 9, 12, 15, 18, and 21 for 1st instar *H. axyridis* larvae; 12, 18, 24, 30, and 36 for 2nd instar *H. axyridis* larvae; 15, 30, 45, 60, and 75 for 3rd instar *H. axyridis* larvae; and 40, 70, 100, 130, and 160 per cup for 4th-instar larvae and female adults (listed in Table S1). Each experimental condition was replicated 4 times. After 24 h, the remaining *R. nymphaeae* nymphs inside individual cups were recorded. A control group was also established at the same prey density without predators, and no natural mortality rates were recorded.

### 2.3. Functional Response Assays

To assess the impact of predators on their prey, we followed the instructions in the “FRAIR” package (v0.5.100) in R Studio [29]. We first visually inspected the data, which led to the rejection of the type I functional response. Then, we used Holling’s type II functional response model for the type II functional response [24,30], which is given by the equation:

*N_a_* = *a*
*N_t_T*/(1 + *aT_h_N_t_*)

where *N_a_* is the number of consumed prey, *N_t_* is the initial prey density, *a* is the attack rate, and *T_h_* is the experimental period (days).

For the evaluation of the foraging strategy, we employed the type III functional response model, represented by the equation [31]:

*N_a_* = *a*′*exp* (−*b/N_t_*)

Here, the attack rate (*a*) is assumed to vary with prey density, and *a*′ is the maximum predacious number when the prey density approaches infinity. The other parameters are the same as in the type II functional response model.

To compare the type II and III functional response models and select the best one, we used the approach proposed by Okuyama (2013) [32]. This involved employing the Akaike information criterion (AIC) to compare the fitted models. The best model is the one with the lowest AIC. Additionally, we used a polynomial logistic function of the proportion of prey consumed (*Na*/*N*_0_) at lower prey densities to better distinguish slight differences in curve shape between type II and III models, as compared to a non-linear curve [33]:

$$\frac{N_{e}}{N_{0}}=\frac{\exp\;(P_{0}+P_{1}N_{0}+P_{2}N_{0}^{2})}{1+\exp\;(P_{0}+P_{1}N_{0}+P_{2}N_{0}^{2})}$$

where *N_e_* is the number of prey consumed, *N*_0_ is the initial prey density, and *P*_0_, *P*_1_, and *P*_2_ are the constant, linear, and quadratic coefficients.

The sign and significance of these coefficients determine the type of functional response. A significant negative linear coefficient (*P*_1_ < 0) suggests a type II functional response (where proportional prey consumption decreases as prey density increases). On the other hand, significant positive linear and negative quadratic coefficients (*P*_1_ > 0 and *P*_2_ < 0) indicate a type III functional response characterized by an initial increase and a subsequent decrease in proportional prey consumption.

To compare the fitted coefficients, 95% confidence intervals (CIs) were generated using nonparametric bootstrapping. Parameters with non-overlapping 95% CIs were considered significantly different. Functional response curves were plotted with their respective 95% CIs.

The relationship between the search effect (*S*) and prey density (*N_t_*) was fitted by the formula [34]:

*S* = *a*/(1 + *aT_h_N_t_*)

where *S* represents the search effect value, and parameters *a* and *T_h_* are the attack rate and handling time of the type II functional response model.

### 2.4. Effects of H. axyridis Density on Predatory Response under Constant Ladybird-to-Aphid Ratio

In the experiment, the ambient conditions matched those previously described. The experiment involved introducing 1, 2, 3, 4, and 5 natural enemies per cup in equal proportions, with a predator-to-prey ratio of 1:20 for 1st instar *H. axyridis* larva, 1:50 for 2nd and 3rd instar *H. axyridis* larva, and 1:100 for 4th-instar larva and adult (list in Table S2). Before the experiment, all predators were starved for 24 h. Predators and prey were then placed into each cup, and after 24 h, the number of surviving prey was counted without replacing any consumed prey. Each developmental stage was replicated four times in the experiment.

According to the Hassell–Varley predation model [35], the intraspecific interference can be represented as follows:

*E* = *qP^−m^*

Here, *E* represents the average predation rate, *q* is the predation rate when the number of predators is one, *P* is the density of prey, and *m* is the intraspecific interference index.

Additionally, the scrambling competition intensity (*I*) can be represented by the following equation:

*I* = (*E*_1_ − *E_p_*)/*E*_1_

where *I* represents the scrambling competition intensity, *E*_1_ is the search efficiency of a single predator, and *E_p_* is the estimated search efficiency of *P* number of predators.

### 2.5. Effects of H. axyridis Density on Predatory Response under Constant Aphid Density

To study the competition and mutual interference within species of *H. axyridis*, we placed varying numbers of predators (1, 2, 3, 4, and 5 larvae per cup) and a constant number of prey (50 for 1st instar *H. axyridis* larva, 100 for 2nd and 3rd-instar *H. axyridis* larva, 500 for 4th-instar larva and adult) into each cup as specified in Table S3. After 24 h, the predation rates were recorded. This process was repeated 5 times.

The experimental data were analyzed using Watt’s interference function [36]:

*A* = *QP^−m^*

where *A* represents the average predation number, *Q* is the maximum predation number when the predators’ number is one, *P* is the density of prey, and *m* is the intraspecific competition parameter.

### 2.6. Statistical Analysis

The data analysis was conducted using SPSS 20.0 software. Fitting and correlation analysis were performed on the functional response model. The data on proportionate prey consumption underwent the Kruskal–Wallis test (nonparametric ANOVA) to assess significant effects. Dunn’s multiple comparison tests were then used to differentiate between group means (*p* < 0.05). The “FRAIR” package (v0.5.100) was utilized to estimate “*a*” and “*Th*” parameters and their standard errors. All data visualization was created in R using R Studio and the “FRAIR” package [29].

## 3. Results

### 3.1. Functional Response

**Figure 1:**
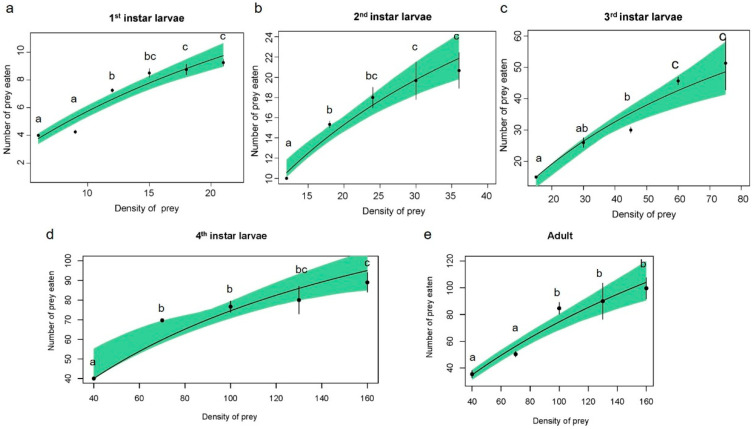
Type II functional response curves of *Harmonia axyridis:* (**a**) 1st-instar larvae; (**b**) 2nd-instar larvae; (**c**) 3rd-instar larvae; (**d**) 4th-instar larvae; and (**e**) adults when preying on *Rhopalosiphum nymphaeae* nymphs. Dots represent the average amount eaten, and bars represent the respective standard errors. Lines were predicted using Holling’s disc equation. Shaded areas represent bootstrapped 95% confidence intervals (95% CI). Note that vertical and horizontal axis scales are not the same across the different stages of *H. axyridis*. Different letters used in the graph indicate significant differences at *p* < 0.05.

The type II and type III functional responses were modeled and presented in Table 1 and Table S4, respectively. The type II functional response showed lower AIC values than the type III functional response for all developmental stages except for the second-instar larvae (Table 2). Additionally, the logistic regression of prey consumption derived linear coefficients between the initial aphid densities offered and the proportion of aphids consumed (Figure 1, Table 3). Based on the coefficients, type II functional response is a suitable model for fitting the predation ability of *H. axyridis* on *R. nymphaeae* at all developmental stages.

### 3.2. Predation Ability and Attack Rate of H. axyridis at Different Development Stages on R. nymphaeae Nymphs

The predation rate of *H. axyridis* on *R. nymphaeae* nymphs significantly increased as prey density increased (Figure 1), which was observed in the first instar (F = 24.3; df = 5, 18; *p* < 0.01), second instar (F = 11.9; df = 4, 10; *p* < 0.01), third instar (F = 14.1; df = 4, 10; *p* < 0.01), fourth instar (F = 21.2; df = 4, 10; *p* < 0.01) larva, and adult (F = 13.9; df = 4, 10; *p* < 0.01).

Adults and fourth-instar larvae of *H. axyridis* consumed significantly more aphids than younger instar larvae (Figure 1). The predation ability (*a*/*T_h_*) of ladybirds increased with the growth stage, reaching a maximum of 288.97 in adults (Table 4). Moreover, the estimated parameters for type II functional response revealed that the attack rate (*a*) increased with the growth stage, while both the adult and fourth-instar larvae exhibited the highest attack rate (*p* > 0.05). The handling time (*T_h_*) of the predator decreased as the growth stages advanced. The theoretical maximum predation rate (1/*T_h_*) of adults reached a maximum of 298.11 aphids per day (Table 1). Consequently, the predation ability of adults was superior to that of the other stages. Modeling of the type III functional response also predicted similar results for handling time as the type II functional response (Table S4).

### 3.3. Search Effects of H. axyridis on R. nymphaeae

**Figure 2:**
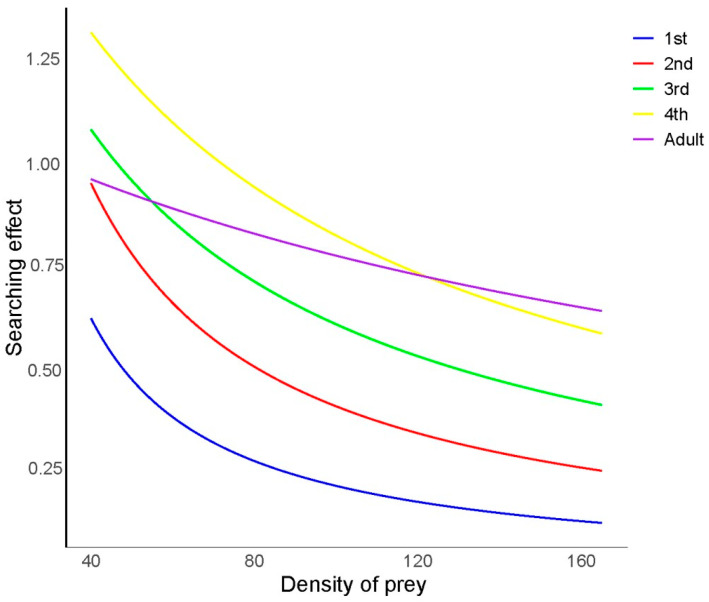
Search effect of *Harmonia axyridis* at different developmental stages on *Rhopalosiphum nymphaeae* nymphs.

As illustrated in Figure 2, the search effects of *H. axyridis* at different developmental stages on *R. nymphaeae* were investigated. The search effect value (*S*) of *H. axyridis* at each developmental stage decreased gradually as the prey density increased. Additionally, at the same density of *R. nymphaeae*, the search effect value (*S*) of *H. axyridis* increased from the first-instar larvae to the fourth-instar larvae. Notably, the fourth-instar larvae exhibited the highest search effect value (*S*) at low initial prey densities (*N_t_* < 124), while adults showed the highest search effect value (*S*) at higher prey densities (*N_t_* > 124), which may be attributed to the shorter handling time of adults.

### 3.4. Intraspecific Interference Influence on the Predation Ability of H. axyridis

When the number of predators was increased, the number of prey consumed by *H. axyridis* was significantly affected across all developmental stages: first instar (F = 44.8; df = 4, 10; *p* < 0.01), second instar (F = 13.9; df = 4, 10; *p* < 0.01), third instar (F = 143.9; df = 4, 10; *p* < 0.01), fourth instar (F = 176.1; df = 4, 10; *p* < 0.01) larvae, and adults (F = 40.8; df = 4, 10; *p* < 0.01) while holding the ladybird-to-aphid ratio constant.

**Figure 3:**
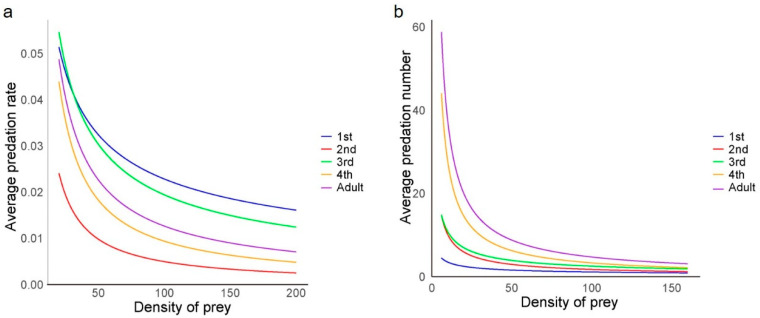
Model equation curves of (**a**) intraspecific interference and (**b**) self-interference among the *Harmonia axyridis* at each developmental stage. Lines were predicted using Hassell–Varley and Watt’s interference function, respectively. ANOVA was used to assess the effect of prey density on predation across different developmental stages.

After applying the Hassell–Varley model, it was observed that the predation rate (*E*) decreased with an increasing number of *H. axyridis* while maintaining the same ladybird-to-aphid population ratio (Figure 3a). This indicated intraspecific competition at all developmental stages of the ladybird, affecting the predation of individuals. The minimum intraspecific interference coefficient (*m*) was 0.504 for first-instar larvae, while the maximum interference coefficient was 0.982 for second-instar larvae (Table 4). Additionally, second-instar larvae exhibited the lowest average predation rate (*E*) at different population densities (Figure 3a), suggesting that they were strongly affected by intraspecific interference during predation. Throughout the experiment, no instances of cannibalism were observed.

The scrambling competition intensity (*I*) among first-instar larvae to adults is shown in Table 5. When there was only one predator, there was no intraspecific interference during predation (*I* = 0). However, as the density of predators increased, competition among predators intensified, and the value of competition intensity (*I*) also rose progressively. An analysis of the relationship between the logarithm of predator density and predation activity rate revealed a positive correlation. This analysis showed a strong linear relationship between the latter and the former (Table 5).

### 3.5. Self-Interference Influences the Predation of H. axyridis

With a constant prey population size, the population size of *H. axyridis* had a significant effect on the number of prey consumed by *H. axyridis* larvae at the first instar (F = 13.9; df = 4, 10; *p* < 0.01), second instar (F = 5.0; df = 4, 10; *p* < 0.01), third instar (F = 9.8; df = 4, 10; *p* < 0.01), and fourth instar (F = 1.6; df = 4, 10; *p* < 0.01) stages. However, there was no significant difference in predation ability for the adult ladybirds exhibited as the population size increased (F = 1.3; df = 4, 10; *p* = 0.335). The average predation number (*A*) decreased with an increase in the number of predators (Figure 3b), indicating the self-interference influence among ladybirds. According to Watt’s interference model, the maximum predation number (*Q*) tended to increase with age, with the lowest *Q* value for the first-instar larvae and the highest for the adult stage, reaching a maximum of 293.4. The intraspecific competition parameter (*m*) also varied across the different larval stages, while the minimum value was 0.494 for first-instar larvae, and the maximum value was 0.920 for fourth-instar larvae (Table 6).

## 4. Discussion

The functional response is a crucial criterion for assessing the predation efficiency of predators on specific prey [37]. Numerous studies have utilized functional response experiments to investigate the predatory behavior of *H. axyridis* against various agricultural pests [19,38]. However, until now, no research has been available on the predation potential of *H. axyridis* on *R. nymphaeae*. Our study evaluated the predation efficiency of *H. axyridis* on *R. nymphaeae* at different predation stages. The comparison of the fitting equations showed that the AIC value of all the developmental stages, except the second larva stage, exhibited a type II functional response. Furthermore, based on logistic regression coefficients, all developmental stages showed a type II functional response. The Type II functional response revealed in our study has been widely reported for various predatory insects, including *H. axyridis* [39,40]. In this Type, the attack rate and handling time are critical parameters of functional response. The attack rate determines the predator’s ability to prey within a given time frame while handling time indicates the duration for a predator to recognize, subdue, attack, and consume a specific prey item. As foraging behavior intensifies, the attack rate increases, leading to a reduction in handling time [41]. In this study, the parameters of functional response were calculated to elucidate how predation efficiency varies with prey densities and predator developmental stages. However, the error bars in the Type II functional response curves exhibited substantial variation (Figure 1), indicating a need for additional replications for functional response experiments.

Our results indicated that fourth-instar larvae and adults of *H. axyridis* beetles were more voracious than other developmental stages. This is likely due to nutritional requirements for development and reproduction. Adult *H. axyridis* beetles exhibited the highest predation ability and predation rate, while the fourth-instar larvae showed the highest search effect at lower prey densities (*N_t_* < 124). In terms of larvae performance, older larvae displayed better predation ability, attack rate, and search effect, which is likely related to higher food and energy demands. In addition, fourth-instar *H. axyridis* larvae were found to perform better when preying on *Aphis glycines* [42]. The poor performance of younger instar larvae in terms of predation ability, attack rate, and search effect may be due to their smaller sizes, slower movements, and lower nutritional requirements, as has been reported [43,44]. These results suggested that fourth-instar larvae and adults of *H. axyridis* would become important biocontrol agents of *R. nymphaeae*.

Previous studies have indicated that as population density increases, predators demonstrate varying levels of intraspecific interference and self-interference when hunting prey [45]. This study analyzed the impact of intraspecific interference and self-interference on the predation ability of *H. axyridis* on *R. nymphaeae* using the Hassell–Varley model and Watt model. The research demonstrates that the search efficiency of *H. axyridis* on *R. nymphaeae* decreased significantly with increasing population density. The scrambling competition intensity formula was used to assess the effect of increased predator density on intraspecific competition. It was observed that adult *H. axyridis* exhibited lower competition intensity (*I*) than second and fourth-instar larvae (Table 5). Furthermore, adults showed no significant difference in predation ability as the population size of *H. axyridis* increased (Table 6). These results indicated that there is relatively mild intraspecific interference in adult *H. axyridis;* hence, increasing their density can be beneficial for field applications and indoor rearing. However, it was noted that adults exhibited a higher level of interference intensity (*m* = 0.897), indicating potential issues when using a large number of *H. axyridis* to control *R. nymphaeae* in a small area [46]. Although indoor study data may not fully capture natural environmental interactions, this information is valuable for understanding interactions among *H. axyridis* populations targeting specific host patches.

Furthermore, it is critical to take into account the effect of temperature when studying functional response. The impact of temperature on the feeding behavior of *H. axyridis* has been observed when it feeds on different prey species such as *Myzus persicae* [40], *Spodoptera litura* [47], and *Acyrthosiphon pisum* [19]. Warmer temperatures can promote the development of *H. axyridis*; however, temperatures above 35 °C can disrupt its life cycle, affecting egg hatching. On the other hand, aphids show a less pronounced response to temperature changes. Considering that some regions in the tropics and Asia experience temperatures exceeding 35 °C during the summer months [48], releasing this predator in such conditions may compromise its effectiveness in controlling *R. nymphaeae* populations, as it could adversely affect the predator’s development and biology. Therefore, it is essential to conduct studies under greenhouse or field conditions to understand the feeding behavior of *H. axyridis* and design effective release strategies for this promising predator to suppress *R. nymphaeae* populations.

In conclusion, this study has preliminarily clarified the functional response and numerical response of *H. axyridis* on *R. nymphaeae* nymphs and found that *H. axyridis* had biological control potential against *R. nymphaeae*. The present results indicated that *H. axyridis* is expected to be a sustainable biocontrol agent for pond garden pests.

## Figures and Tables

**Table 1:** Theoretical values of parameters reflecting predation ability for type II function response.

Ladybird Stage	Parameter	Mean ± SE (95% CI)	1/*T_h_*	*a*/*T_h_*	χ^2^
1st instar larva	*a*	0.7372 ± 0.0822 (0.553–0.866)	24.69	18.20	0.3906
	*T_h_*	0.0405 ± 0.0040 (0.016–0.055)			
2nd instar larva	*a*	1.0680 ± 0.2191 (0.595–1.998)	53.34	57.11	0.2717
	*T_h_*	0.0187 ± 0.0010 (0.0158–0.0225)			
3rd instar larva	*a*	1.1570 ± 0.1354 (0.782–1.810)	103.58	119.28	1.4010
	*T_h_*	0.0097 ± 0.0005 (0.0020–0.0157)			
4th instar larva	*a*	1.3878 ± 0.5010 (0.839–1.769)	163.55	227.51	2.0017
	*T_h_*	0.0061 ± 0.0006 (0.0024–0.0133)			
Adult	*a*	0.9825 ± 0.0843 (0.6132–1.2182)	298.11	288.97	2.2427
	*T_h_*	0.0034 ± 0.0015 (0.0014–0.017)			

Values in parentheses are 95% CIs. Means ± standard errors within columns show effect of developmental stage on attack rate.

**Table 2:** Akaike information criterion (AIC) for the two candidate functional response models for each developmental stage.

Ladybird Stage	Type II	Type III
1st instar larva	73.00	73.67
2nd instar larva	92.52	80.08
3rd instar larva	668.78	857.56
4th instar larva	674.30	946.79
Adult	2160.99	2213.39

**Table 3:** Estimates and respective standard error (SE) of the linear coefficient of logistic regression analysis for aphids consumed by ladybirds.

Ladybird Stage	Estimate	SE	Z-Value	*p*-Value
1st instar larva	−0.78	2.39 × 10^−4^	−0.33	0.74
2nd instar larva	−0.52	2.83 × 10^−4^	0.18	0.85
3rd instar larva	−1.12	2.09 × 10^−4^	−0.54	0.59
4th instar larva	−0.99	2.89 × 10^−4^	−0.34	0.73
Adult	−0.11	0.39 × 10^−3^	−0.28	0.78

**Table 4:** Theoretical values of parameters reflecting interference level for Hassell–Varley model.

Ladybird Stage	Model Equation	*q*	m	R^2^	χ^2^
1st larva	*E* = 0.2326*P*^−0.504^	0.2326	0.504	0.986	0.0012
2nd larva	*E* = 0.4565*P*^−0.982^	0.4565	0.982	0.963	0.0443
3rd larva	*E* = 0.3751*P*^−0.643^	0.3751	0.643	0.784	0.0310
4th larva	*E* = 0.7765*P*^−0.959^	0.7765	0.959	0.976	0.4429
Adult	*E* = 0.5999*P*^−0.838^	0.5999	0.838	0.989	1.95

**Table 5:** Theoretical values of parameters and functions reflecting predation rate scrambling competition.

Ladybird Stage	Number	*I*	Model Equation	R^2^
1st instar larva	1	0.0000	*I* = 0.7899log*P*+0.0287	0.974
	2	0.3214		
	3	0.4127		
	4	0.4688		
	5	0.5829		
2nd instar larva	1	0.0000	*I* = 1.1155log*P*+0.1249	0.850
	2	0.6487		
	3	0.7271		
	4	0.7650		
	5	0.8030		
3rd instar larva	1	0.0000	*I* = 0.9776log*P*−0.0897	0.877
	2	0.0316		
	3	0.3958		
	4	0.5194		
	5	0.6375		
4th instar larva	1	0.0000	*I =* 1.1082log*P*+0.1102	0.878
	2	0.6062		
	3	0.7109		
	4	0.7406		
	5	0.7975		
Adult	1	0.0133	*I =* 1.7777log*P*−0.8417	0.9951
	2	−0.3167		
	3	0.0341		
	4	0.2067		
	5	0.4053		

**Table 6:** Theoretical values of parameters and functions reflecting interference level for Watt’s model.

Ladybird Stage	Functional Response Equation	*Q*	*m*	R^2^	χ^2^
1st instar larva	*A* = 10.853*P*^−0.494^	10.853	0.494	0.963	0.1287
2nd instar larva	*A* = 58.547*P*^−0.765^	58.54	0.765	0.997	0.1143
3rd instar larva	*A* = 45.969*P*^−0.631^	45.969	0.631	0.968	0.7139
4th instar larva	*A* = 229.530*P*^−0.920^	229.530	0.920	0.983	5.3019
Adult	*A* = 293.400*P*^−0.897^	293.400	0.897	0.992	1.7062

## Data Availability

All data included in this study are available upon request by contact with the corresponding author.

## Supplementary Materials

The following supporting information can be downloaded at: https://www.mdpi.com/article/10.3390/insects15090633/s1, Table S1: Density of *Rhopalosiphum nymphaeae* offered to *Harmonia axyridis* for each stage. Table S2: Density ratio of *Rhopalosiphum nymphaeae* and *Harmonia axyridis* for each stage. Table S3: Density of *Rhopalosiphum nymphaeae*: *Harmonia axyridis* for each stage. Table S4: Theoretical values of parameter reflecting predation ability for type III function response.

## References

[1] Mishra R., Jha B., Jha V., Singh S., Mahato A. (1992). Insect associations of Euryale ferox Salisb. in the ponds of Darbhanga, North Bihar. J. Freshw. Biol..

[2] Holman J. (2009). Host Plant Catalog of Aphids Palaearctic Region.

[3] Mille C., Jourdan H., Cazères S., Maw E., Foottit R. (2020). New data on the aphid (Hemiptera, Aphididae) fauna of New Caledonia: Some new biosecurity threats in a biodiversity hotspot. ZooKeys.

[4] Assour H.R., Ashman T.L., Turcotte M.M. (2024). Neopolyploidy-induced changes in giant duckweed (Spirodela polyrhiza) alter herbivore preference and performance and plant population performance. Am. J. Bot..

[5] Kumar D.M., Kumar P., Ramya N., Chakraborty A., Dey J. (2021). Plant Health Issues in Fox Nut/Makhana (*Euryale ferox*): An Agronomic Perspective. J. Plant Health Issues.

[6] Wang Y., Xu S. (2024). A high-quality genome assembly of the waterlily aphid *Rhopalosiphum nymphaeae*. Sci. Data.

[7] Seth M., Raychaudhuri S. (1973). Further studies on a new mosaic disease of brinjal (*Solanum melongena* L.). Proc. Indian Natl. Sci. Acad. B.

[8] Pettet A., Pettet S.J. (1970). Biological Control of *Pistia stratiotes* L. in Western State, Nigeria. Nature.

[9] Center T.D. (2002). Insects and Other Arthropods That Feed on Aquatic and Wetland Plants.

[10] Shen Y., Zhang Y., Guo H., Zhu S., Xu W. (2016). Insecticidal activity of three pesticides against *Rhopalosiphum nymphaeae* in lotus and their safety to crops. Plant Prot..

[11] Qu C., Mu C., Zhu H., Li B., Li F., Luo C. (2022). Laboratory toxicity and control effect of seven insecticides to *Rhopalosiphum nymphaeae*. China Plant Prot..

[12] Sun H., Li H., Zhang X., Liu Y., Chen H., Zheng L., Zhai Y., Zheng H. (2023). The honeybee gut resistome and its role in antibiotic resistance dissemination. Integr Zool..

[13] Intisar A., Ramzan A., Sawaira T., Kareem A.T., Hussain N., Din M.I., Bilal M., Iqbal H.M.N. (2022). Occurrence, toxic effects, and mitigation of pesticides as emerging environmental pollutants using robust nanomaterials—A review. Chemosphere.

[14] Zhang Y., Xu H., Tu C., Han R., Luo J., Xu L. (2024). Enhanced capacity of a leaf beetle to combat dual stress from entomopathogens and herbicides mediated by associated microbiota. Integr. Zool..

[15] Ma M., Luo J., Li C., Eleftherianos I., Zhang W., Xu L. (2023). A life-and-death struggle: Interaction of insects with entomopathogenic fungi across various infection stages. Front. Immunol..

[16] Gao G., Liu S., Feng L., Wang Y., Lu Z. (2020). Effect of temperature on predation by *Harmonia axyridis* (Pall.) (Coleoptera: Coccinellidae) on the walnut aphids *Chromaphis juglandicola* Kalt. and *Panaphis juglandis* (Goeze). Egypt. J. Biol. Pest Control.

[17] Raak-van den Berg C.L., Hemerik L., van der Werf W., de Jong P.W., van Lenteren J.C. (2017). Life history of the harlequin ladybird, *Harmonia axyridis*: A global meta-analysis. BioControl.

[18] Ingels B. (2013). Intraguild interactions between the Harlequin Ladybird *Harmonia axyridis* and Non-Coccinellid Aphidophagous Predators. Ph.D. Thesis.

[19] Islam Y., Shah F.M., Rubing X., Razaq M., Yabo M., Xihong L., Zhou X. (2021). Functional response of *Harmonia axyridis* preying on *Acyrthosiphon pisum* nymphs: The effect of temperature. Sci. Rep..

[20] DeLong J.P., Uiterwaal S.F. (2022). Predator functional responses and the biocontrol of aphids and mites. BioControl.

[21] Solomon M.E. (1949). The natural control of animal populations. J. Anim. Ecol..

[22] Holling C.S. (1959). The components of predation as revealed by a study of small-mammal predation of the european pine sawfly. Can. Entomol..

[23] Jeschke J.M., Kopp M., Tollrian R. (2004). Consumer-food systems: Why type I functional responses are exclusive to filter feeders. Biol. Rev. Camb. Philos. Soc..

[24] Holling C.S. (1959). Some Characteristics of simple types of predation and parasitism. Can. Entomol..

[25] Pervez A., Omkar (2005). Functional responses of coccinellid predators: An illustration of a logistic approach. J. Insect Sci..

[26] Park T. (1962). Beetles, competition, and populations. Science.

[27] Skalski G.T., Gilliam J.F. (2001). Functional responses with predator interference: Viable alternatives to the Holling type II model. Ecology.

[28] Kratina P., Vos M., Bateman A., Anholt B.R. (2009). Functional responses modified by predator density. Oecologia.

[29] Pritchard D.W., Paterson R.A., Bovy H.C., Barrios-O’Neill D. (2017). frair: An R package for fitting and comparing consumer functional responses. Methods Ecol. Evol..

[30] Wu K., Shen C., Gong P. (2004). Equation of predator functional response and estimation of the parameters in it. Kun Chong Zhi Shi.

[31] Wang S., Xia C. (1988). The new functional response model of type III Holling equation. Chin. J. Ecol..

[32] Okuyama T. (2013). On selection of functional response models: Holling’s models and more. BioControl.

[33] Juliano S. (2001). Nonlinear curve fitting: Predation and functional response curves. Design and Analysis of Ecological Experiments.

[34] Ding Y. (1994). Mathematical Ecology of Insects.

[35] Hassell M.P. (1969). A Population model for the interaction between *Cyzenis albicans* (Fall.) (Tachinidae) and *Operophtera brumata* (L.) (Geometridae) at Wytham, Berkshire. J. Anim. Ecol..

[36] Watt K.E.F. (1959). A Mathematical model for the effect of densities of attacked and attacking species on the number attacked. Can. Entomol..

[37] Real L.A. (1977). The kinetics of functional response. Am. Nat..

[38] Feng Y., Li Y.D., Liu Z.G., Yu X.L., Zhu G.X., Keller M., Liu T.X. (2019). Behavioural patterns and functional responses of a generalist predator revealed using automated video tracking. Pest Manag. Sci..

[39] Hassanpour M., Mohaghegh J., Iranipour S., Nouri-Ganbalani G., Enkegaard A. (2011). Functional response of *Chrysoperla carnea* (Neuroptera: Chrysopidae) to *Helicoverpa armigera* (Lepidoptera: Noctuidae): Effect of prey and predator stages. Insect Sci..

[40] Yu X.-L., Zhang Y.-J., Zuo J.-F., Luo X., Zhang L., Danzeng Z.-M., Wang B., Xia P.-L., Zhang S.-Z., Liu T.-X. (2023). Rising temperatures affect the interspecific interference competition between *Harmonia axyridis* and *Propylea japonica*, and their predation rate on *Myzus persicae*. J. Pest Sci..

[41] Abracos-Duarte G., Ramos S., Valente F., Borges da Silva E., Figueiredo E. (2021). Functional response and predation rate of *Dicyphus cerastii* Wagner (Hemiptera: Miridae). Insects.

[42] Xue Y., Bahlai C.A., Frewin A., Sears M.K., Schaafsma A.W., Hallett R.H. (2009). Predation by *Coccinella septempunctata* and *Harmonia axyridis* (Coleoptera: Coccinellidae) on *Aphis glycines* (Homoptera: Aphididae). Environ. Entomol..

[43] Moura R., Garcia P.c., Cabral S., Soares A.O. (2006). Does pirimicarb affect the voracity of the euriphagous predator, *Coccinella undecimpunctata* L. (Coleoptera: Coccinellidae)?. Biol. Control.

[44] Islam Y., Shah F.M., Shah M.A., Musa Khan M., Rasheed M.A., Ur Rehman S., Ali S., Zhou X. (2020). Temperature-dependent functional response of *Harmonia axyridis* (Coleoptera: Coccinellidae) on the Eggs of *Spodoptera litura* (Lepidoptera: Noctuidae) in Laboratory. Insects.

[45] Jia J., Fu Y., Zhang F., Liang M., Chen J. (2019). Effects of temperature on predatory functional responses of *Neoseiuius californicus* to *Eutetranychus orientalis*. Chin. J. Biol. Control.

[46] Tripathi J.P., Jana D., Vyshnavi Devi N.S.N.V.K., Tiwari V., Abbas S. (2020). Intraspecific competition of predator for prey with variable rates in protected areas. Nonlinear Dyn..

[47] Islam Y., Shah F.M., Guncan A., DeLong J.P., Zhou X. (2022). Functional response of *Harmonia axyridis* to the larvae of *Spodoptera litura*: The combined effect of temperatures and prey instars. Front. Plant Sci..

[48] Majumdar P., Debnath S., Sarkar S., Ghosh U. (2021). The complex dynamical behavior of a prey-predator model with Holling type-III functional response and non-linear predator harvesting. Int. J. Model. Simul..

