# Supplementary material for: Functional and Numerical Responses of Harmonia axyridis (Coleoptera: Coccinellidae) to Rhopalosiphum nymphaeae (Hemiptera: Aphididae) and Their Potential for Biological Control
Source: Insects. 2024 Aug 23;15(9):633. doi: 10.3390/insects15090633 (PMC11432611; doi:10.3390/insects15090633)
Supplement: Supplementary file 1 [file insects-15-00633-s001.zip › insects-3145880-supplementary.pdf]

Table S1. Density of *Rhopalosiphum nymphaeae* offered to *Harmonia axyridis* for each stage.

Ladybird stage	Density					
1st	6	9	12	15	18	21
2nd	12	18	24	30	36	
3rd	15	30	45	60	75	
4th	40	70	100	130	160	
Adult	40	70	100	130	160	

Table S2. Density ratio of *Rhopalosiphum nymphaeae* and *Harmonia axyridis* for each stage.

Ladybird stage	Density of *H. axyridis*: *R. nymphaeae*				
1st	1:20	2:40	3:60	4:80	5:100
2nd	1:50	2:100	3:150	4:200	5:250
3rd	1:50	2:100	3:150	4:200	5:250
4th	1:100	2:200	3:300	4:400	5:500
Adult	1:100	2:200	3:300	4:400	5:500

Table S3. Density of *Rhopalosiphum nymphaeae*: *Harmonia axyridis* for each stage.

Ladybird stage	Density of *H. axyridis*: *R. nymphaeae*				
1st	1:50	2:50	3:50	4:50	5:50
2nd	1:100	2:100	3:100	4:100	5:100
3rd	1:100	2:100	3:100	4:100	5:100
4th	1:500	2:500	3:500	4:500	5:500
Adult	1:500	2:500	3:500	4:500	5:500

Table S4. Theoretical values of parameter reflecting predation ability for type III function response.

Ladybird stage	Functional Response Equation	Parameter	Estimate	R	χ^2
1st	N_a=13.35·exp$^{(-7.91/Nt)}$	*a'*	13.35	0.930	0.4222
		b	7.91		
2nd	N_a=30.63·exp$^{(-13.16/Nt)}$	*a'*	30.63	0.999	0.0500
		b	13.16		
3rd	N_a=59.42·exp$^{(-21.65/Nt)}$	*a'*	59.42	0.887	3.0600
		b	21.65		
4th	N_a=115.38·exp$^{(-41.09/Nt)}$	*a'*	115.38	0.992	0.7178
		b	41.09		
Adult	N_a=137.88·exp$^{(-57.06/Nt)}$	*a'*	137.88	0.943	2.7210
		b	57.06		
